# Behavioral Procrastination and Heart Age Acceleration in a Large Occupational Cohort

**DOI:** 10.3390/jcm15135190

**Published:** 2026-07-02

**Authors:** Manuel Sarmiento Cruz, Pedro Juan Tárraga López, Mónica Silu Piña Dabreu, Lluis Rodas Cañellas, Ángel Arturo López-González, José Ignacio Ramírez-Manent

**Affiliations:** 1 Primary Care, Balearic Islands Health Service, 07010 Palma, Spain; manuel.sarmiento@ibsalut.es (M.S.C.);; 2 Faculty of Medicine, University of Castilla La Mancha (UCLM), 02008 Albacete, Spain; 3 ADEMA-University School, University of the Balearic Islands, 07009 Palma, Spain

**Keywords:** procrastination, cardiovascular diseases, vascular aging, occupational health, life style, risk factors

## Abstract

**Background:** Behavioral procrastination has been increasingly recognized as a maladaptive self-regulatory pattern associated with unhealthy lifestyle behaviors, psychological stress, and adverse cardiometabolic profiles. However, its relationship with accelerated cardiovascular aging remains poorly understood. This study aimed to evaluate the association between behavioral procrastination and heart age acceleration in a large occupational cohort of Spanish workers. **Methods:** A multicenter cross-sectional study was conducted including 92,184 actively employed Spanish workers undergoing routine occupational health examinations between 2021 and 2024. Behavioral procrastination was assessed using the Pure Procrastination Scale-9 (PPS-9). Estimated heart age and heart age acceleration were calculated using a cardiovascular risk-factor-based algorithm. Multivariable linear and logistic regression analyses were performed to evaluate associations between procrastination score, continuous heart age acceleration, and accelerated cardiovascular aging phenotypes after adjustment for demographic, lifestyle, anthropometric, and cardiometabolic variables. Restricted cubic spline analyses and sex-stratified analyses were additionally conducted. **Results:** Higher procrastination levels were associated with progressively worse cardiometabolic and cardiovascular aging profiles. Mean heart age acceleration increased from −3.1 ± 6.0 years in participants with very low procrastination to 14.0 ± 6.4 years in those with very high/chronic procrastination (*p* < 0.001). The prevalence of accelerated cardiovascular aging (>0 years) increased from 27.2% to 94.2% across increasing procrastination categories, whereas severe accelerated cardiovascular aging (≥10 years) increased from 1.7% to 75.6% (both *p* < 0.001). In fully adjusted multivariable analyses, each 5-point increase in PPS-9 score was associated with a 0.50-year increase in heart age acceleration (B = 0.50; 95% CI 0.48–0.52; *p* < 0.001). Participants with very high/chronic procrastination exhibited significantly higher odds of accelerated cardiovascular aging (OR 1.89; 95% CI 1.65–2.18) and severe accelerated cardiovascular aging (OR 2.51; 95% CI 2.16–2.92). Associations were significantly stronger among women (*p*-interaction < 0.001). Findings remained robust in sensitivity analyses excluding participants with diabetes mellitus. **Conclusions:** Behavioral procrastination was associated with higher estimated heart age acceleration and less favorable cardiovascular aging profiles in this large occupational cohort. Higher procrastination severity was consistently related to greater estimated heart age acceleration and a higher prevalence of cardiovascular aging phenotypes, even after extensive multivariable adjustment. These findings indicate that higher procrastination levels were associated with less favorable cardiovascular aging profiles beyond traditional biomedical risk factors. However, given the cross-sectional design, no conclusions regarding causality or temporality can be drawn.

## 1. Introduction

Cardiovascular disease remains the leading cause of mortality worldwide and continues to represent a major challenge for preventive medicine despite substantial advances in cardiovascular risk management during recent decades [1]. Although classical risk factors such as hypertension, diabetes mellitus, dyslipidemia, obesity, smoking, and sedentary lifestyle remain central to cardiovascular prevention strategies, increasing attention has recently been directed toward the concept of accelerated cardiovascular aging as a more integrative marker of cumulative cardiovascular risk burden [2,3].

Cardiovascular aging reflects the progressive structural and functional deterioration of the vascular system resulting from the interaction between biological aging, metabolic dysfunction, chronic inflammation, oxidative stress, and long-term exposure to adverse lifestyle and psychosocial factors [4]. Importantly, individuals of the same chronological age may exhibit markedly different degrees of vascular aging, suggesting that behavioral and environmental determinants substantially influence cardiovascular aging trajectories [5].

Within this context, estimated heart age has emerged as a clinically useful and intuitive approach for evaluating cardiovascular aging and communicating cardiovascular risk [6]. Heart age models integrate multiple cardiovascular risk factors to estimate the biological age of the cardiovascular system relative to chronological age, thereby allowing identification of individuals with premature cardiovascular aging phenotypes [7]. Previous studies have demonstrated that excess heart age is associated with increased risk of cardiovascular disease, subclinical vascular dysfunction, and premature mortality [8,9].

Beyond traditional biomedical risk factors, psychosocial and behavioral determinants are increasingly recognized as important contributors to cardiovascular health [10]. Chronic psychological stress, emotional dysregulation, sleep disturbances, maladaptive coping strategies, and impaired self-regulation have all been associated with endothelial dysfunction, autonomic imbalance, systemic inflammation, and adverse cardiometabolic outcomes [11,12]. In parallel, behavioral medicine research has progressively highlighted the role of self-regulatory dysfunction as a determinant of long-term health behaviors and cardiovascular risk accumulation [13].

Behavioral procrastination is currently understood as a complex self-regulatory failure characterized by the unnecessary delay of intended actions despite anticipating negative consequences [14]. Contemporary models suggest that procrastination is not merely a time-management problem but rather a maladaptive behavioral pattern involving emotional avoidance, impaired executive control, impulsivity, and difficulties prioritizing long-term goals over short-term emotional relief [15,16]. Individuals with elevated procrastination levels frequently exhibit poorer lifestyle behaviors, including reduced physical activity, unhealthy dietary patterns, smoking, sleep disruption, and delayed engagement in preventive healthcare behaviors [17,18,19].

Importantly, many of these behavioral characteristics overlap with mechanisms known to contribute to premature cardiovascular aging. Chronic stress exposure and persistent emotional dysregulation may promote hypothalamic–pituitary–adrenal axis activation, sympathetic overactivity, inflammation, oxidative stress, visceral adiposity accumulation, and vascular dysfunction [20,21]. Furthermore, procrastination has been associated with increased psychological distress, fatigue, poorer perceived health, and adverse cardiovascular risk profiles in previous observational studies [22,23,24]. However, despite these observations, the relationship between behavioral procrastination and accelerated cardiovascular aging remains poorly understood.

Most previous investigations evaluating procrastination and health have focused primarily on academic performance, mental health outcomes, or subjective well-being [14,15,16]. Only a limited number of studies have explored its association with objective cardiometabolic risk markers, and data regarding cardiovascular aging phenotypes are currently lacking [23]. This knowledge gap is particularly relevant because behavioral phenotypes associated with chronic self-regulatory dysfunction may be associated with cardiovascular risk accumulation many years before overt cardiovascular disease becomes clinically manifest.

Occupational populations provide a particularly valuable setting for investigating these associations. Modern work environments frequently combine chronic stress exposure, sedentary behavior, time pressure, sleep disruption, and unhealthy lifestyle habits, all of which may interact with self-regulatory dysfunction and cardiovascular risk progression [25]. Moreover, occupational health cohorts allow large-scale standardized evaluation of anthropometric, biochemical, and behavioral variables under relatively homogeneous clinical conditions.

Therefore, the present study aimed to evaluate the association between behavioral procrastination and accelerated cardiovascular aging in a large occupational cohort of Spanish workers. Specifically, we investigated the relationship between procrastination severity and estimated heart age acceleration while additionally exploring dose–response patterns and sex-specific associations.

## 2. Methods

### 2.1. Study Design and Population

A multicenter cross-sectional observational study was conducted using data obtained from routine occupational health examinations performed between January 2021 and December 2024 in several Spanish regions. The study population consisted of actively employed workers undergoing periodic workplace medical assessments as part of occupational health surveillance programs.

The initial database included 94,327 workers aged between 18 and 69 years. Participants with missing information regarding behavioral procrastination, cardiovascular age estimation, anthropometric measurements, blood pressure, biochemical variables, or lifestyle-related covariates were excluded from the analysis. After application of exclusion criteria, the final analytical sample comprised 92,184 workers.

**Figure 1:**
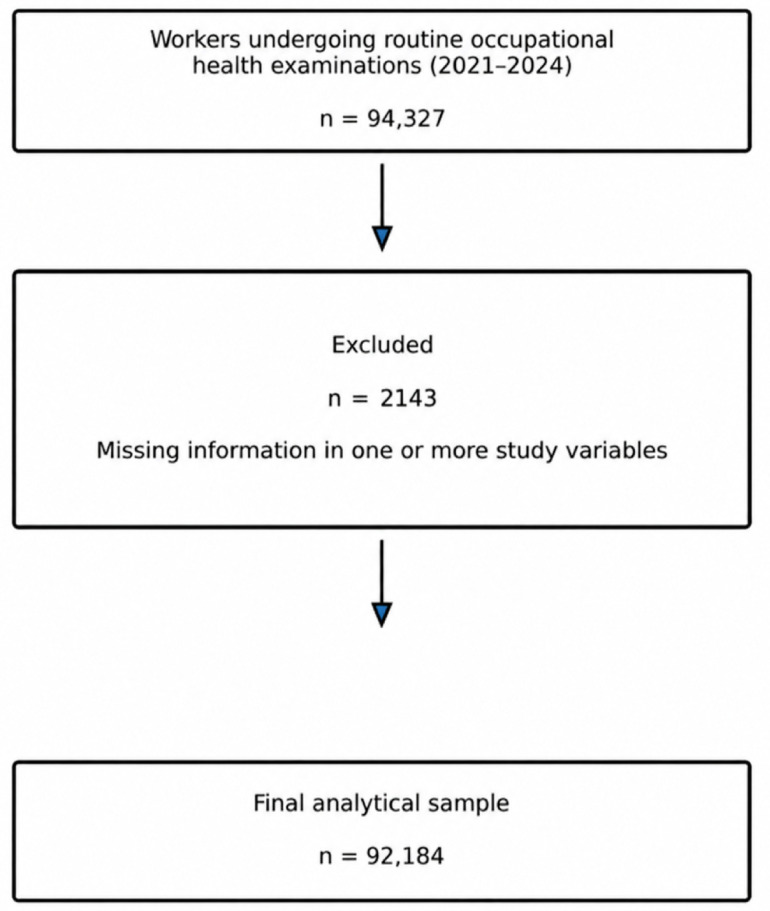
Flow diagram showing participant selection, exclusions due to missing data, and the final analytical sample included in the study.

The participant selection process is summarized in Figure 1.

The study was designed and reported according to current recommendations for observational epidemiological research and cross-sectional studies [26,27].

### 2.2. Ethical Considerations

All participants underwent occupational health assessments as part of routine preventive workplace surveillance and provided consent for the use of anonymized clinical data for research purposes. Personal identifiers were removed before statistical analysis, and all data management procedures complied with current Spanish and European data protection regulations. The study was conducted in accordance with the ethical principles established in the Declaration of Helsinki for research involving human participants [28]. The research protocol was reviewed and approved by the Research Ethics Committee of the Balearic Islands (Comité de Ética de la Investigación de las Islas Baleares, CEI-IB; reference IB 4383/20 PI), which issued a favorable ethical opinion on 25 November 2020.

### 2.3. Assessment of Behavioral Procrastination

Behavioral procrastination was assessed using the Pure Procrastination Scale-9 (PPS-9), a brief self-administered questionnaire designed to measure habitual procrastination behaviors in everyday life. The PPS-9 comprises nine items rated on a five-point Likert scale, yielding a total score ranging from 9 to 45, with higher scores indicating greater behavioral procrastination.

For descriptive analyses, participants were classified into four predefined categories according to their PPS-9 total score: very low/absent procrastination (9–17 points), moderate procrastination (18–26 points), high procrastination (27–35 points), and very high/chronic procrastination (36–45 points). These categories were established a priori to facilitate clinical interpretation and descriptive comparisons across increasing levels of procrastination severity. As universally accepted clinical cut-off values for the PPS-9 are currently lacking, these categories should be considered operational rather than validated diagnostic classifications.

To preserve the full variability of the data and minimize the loss of information associated with categorization, all primary regression analyses were additionally performed using the PPS-9 total score as a continuous variable, allowing the evaluation of dose–response associations with cardiovascular aging phenotypes.

The PPS-9 is a previously validated instrument for assessing behavioral procrastination. In the occupational health database used in the present study, the total PPS-9 score was available, whereas responses to the individual questionnaire items were not retained. Consequently, psychometric re-evaluation of internal consistency, factor structure, and other measurement properties within the current cohort could not be performed. Likewise, item-level analyses and other secondary psychometric assessments were not feasible.

### 2.4. Anthropometric and Clinical Assessment

Anthropometric measurements were obtained by trained occupational health professionals using standardized procedures. Body weight was measured using calibrated electronic scales with participants wearing light clothing and no shoes. Height was measured using a wall-mounted stadiometer. Body mass index (BMI) was calculated as weight in kilograms divided by height in meters squared.

Waist circumference was measured with a non-elastic tape at the midpoint between the lower costal margin and the iliac crest after normal expiration. Waist-to-height ratio was additionally calculated as waist circumference divided by height.

Blood pressure was measured after at least five minutes of seated rest using validated automated sphygmomanometers. When two measurements were available, the mean value was used for analysis. Systolic and diastolic blood pressure were included both as continuous variables and as components of cardiovascular age estimation.

The assessment of central adiposity indicators was performed according to current recommendations emphasizing the clinical relevance of abdominal obesity for cardiovascular risk stratification [29].

### 2.5. Biochemical Measurements

Venous blood samples were obtained after overnight fasting. Laboratory analyses included fasting plasma glucose, total cholesterol, HDL-cholesterol, LDL-cholesterol, and triglycerides. Biochemical determinations were performed in certified laboratories using standardized automated methods with internal quality-control procedures.

All biochemical parameters were expressed in mg/dL and were incorporated into cardiovascular risk estimation algorithms and descriptive cardiometabolic analyses.

### 2.6. Estimation of Cardiovascular Aging

The primary outcome of the study was accelerated cardiovascular aging, assessed using estimated heart age.

Heart age was calculated using the Heart Age Tool developed and validated by López-González et al. [6], which is based on the general cardiovascular risk equations originally developed in the Framingham Heart Study by D’Agostino et al. [8]. The algorithm incorporated age, sex, systolic blood pressure, smoking status, body mass index, diabetes-related variables, and lipid-related parameters to estimate the biological age of the cardiovascular system relative to chronological age. Specifically, the algorithm incorporates chronological age, sex, systolic blood pressure, smoking status, diabetes status, body mass index, total cholesterol, and HDL-cholesterol. Accordingly, the variables embedded within the heart age calculation were chronological age, sex, systolic blood pressure, smoking status, diabetes status, body mass index, total cholesterol, and HDL-cholesterol. The methodological development, calibration procedures, and validation of the algorithm have been described in detail elsewhere [6,7,8]. Because the present study used an externally developed Framingham-based Heart Age tool rather than deriving a new prediction model, discrimination and calibration metrics were not recalculated in the current cohort. The suitability of this approach for the present occupational population is supported by its previous use and validation in Southern European and Spanish working populations [6,7].

Heart age acceleration was calculated as:

Heart age acceleration = Estimated heart age − Chronological age

Positive values indicated that the estimated cardiovascular age exceeded chronological age, reflecting premature cardiovascular aging.

For categorical analyses:

- accelerated cardiovascular aging was defined as heart age acceleration >0 years,
- severe accelerated cardiovascular aging was defined as heart age acceleration ≥10 years.

These definitions were selected to differentiate between mild excess cardiovascular aging and clinically relevant premature cardiovascular aging phenotypes. The conceptual framework was consistent with previous vascular age and cardiovascular risk communication models [30,31,32,33]. Because the complete computational algorithm has been previously published and validated, we have cited the original methodological sources rather than reproducing the full equation within the present manuscript. The online calculator used in the present study implements these previously published Framingham-based equations without modification. Therefore, the coefficients, calibration procedures, and risk functions correspond to those reported in the original methodological publications [6,7,8].

### 2.7. Lifestyle and Sociodemographic Variables

Information regarding smoking status, physical activity, dietary habits, educational level, and occupational characteristics was collected during standardized occupational health interviews.

Smoking status was categorized as current smoker, former smoker, or never smoker. Current smoking was included as a covariate in multivariable analyses because of its established association with cardiovascular aging and vascular dysfunction.

Physical activity was assessed using the International Physical Activity Questionnaire (IPAQ), a validated instrument widely employed in epidemiological studies to estimate habitual physical activity levels [34,35]. Participants were classified according to regular participation in moderate or vigorous leisure-time physical activity based on IPAQ-derived categories.

Dietary habits were evaluated using the Mediterranean Diet Adherence Screener (MEDAS), a validated 14-item questionnaire developed within the PREDIMED study to assess adherence to the Mediterranean dietary pattern [36,37]. According to MEDAS scoring criteria, participants were categorized as having low, moderate, or high adherence to the Mediterranean diet.

Educational level was classified into primary, secondary, and university education. Age and sex were included as core demographic variables in all adjusted analyses.

### 2.8. Statistical Analysis

Continuous variables were expressed as mean ± standard deviation (SD), whereas categorical variables were presented as absolute frequencies and percentages. Normality was assessed using the Kolmogorov–Smirnov test.

Comparisons across procrastination categories were performed using one-way analysis of variance (ANOVA) for continuous variables and chi-square tests for categorical variables.

Behavioral procrastination was analyzed both categorically and continuously. The distribution of chronological age, estimated heart age, heart age acceleration, cardiovascular risk factors, and lifestyle-related variables was evaluated across procrastination categories.

Multivariable linear regression analyses were performed to evaluate the association between behavioral procrastination, measured by the PPS-9, and heart age acceleration as a continuous outcome. Both unstandardized regression coefficients (B) and standardized beta coefficients (β), together with their corresponding 95% confidence intervals (95% CI), were estimated.

Sequential regression models were constructed as follows:

Model 1: unadjusted.
Model 2: adjusted for age and sex.
Model 3: additionally adjusted for educational level, smoking status, physical activity, and adherence to the Mediterranean diet.
Model 4: additionally adjusted for waist-to-height ratio and triglyceride concentration.

Several of the variables included in the sequential models, particularly physical activity, Mediterranean diet adherence, adiposity indicators, and cardiometabolic characteristics, may plausibly act both as confounders and as intermediates within the pathway linking behavioral procrastination and cardiovascular aging. Consequently, the adjusted models should be interpreted as estimates of association after accounting for these factors rather than as estimates of a direct causal effect.

Logistic regression analyses were performed to estimate odds ratios (ORs) and 95% confidence intervals (95% CIs) for accelerated cardiovascular aging and severe accelerated cardiovascular aging according to procrastination category. Because accelerated cardiovascular aging was a common outcome in this study population, ORs were interpreted as measures of association rather than as approximations of relative risks or prevalence ratios.

To provide effect estimates that are more directly interpretable for common outcomes, additional Poisson regression models with robust variance were performed to estimate prevalence ratios (PRs) and corresponding 95% confidence intervals for accelerated cardiovascular aging. The primary inferential analyses were based on the multivariable logistic regression models, whereas PRs were estimated as complementary measures to facilitate interpretation of the magnitude of the associations.

Restricted cubic spline analyses were conducted to explore potential nonlinear dose–response relationships between continuous PPS-9 score and heart age acceleration.

Sex-stratified analyses were performed to evaluate whether associations differed between men and women. Interaction terms between sex and PPS-9 score were additionally tested.

To address potential age-related confounding, additional age-stratified sensitivity analyses were performed. Participants were categorized into four predefined age groups (18–34, 35–44, 45–54, and ≥55 years). The fully adjusted regression model (Model 4) was repeated within each age stratum to evaluate whether the association between PPS-9 score and heart age acceleration persisted among participants of comparable chronological age.

Sensitivity analyses excluding participants with diabetes mellitus were performed to assess the robustness of the findings in a lower-risk preventive population.

Multicollinearity was evaluated using variance inflation factors. Statistical significance was established at a two-sided *p*-value < 0.05. Statistical analyses were performed using SPSS Statistics version 30.0 (IBM Corp., Armonk, NY, USA) and R statistical software (version R 4.6.x) for spline-based analyses and graphical representations.

## 3. Results

### 3.1. Study Population

The final study population included 92,184 active workers. According to behavioral procrastination category, 18,829 participants (20.4%) were classified as having very low/absent procrastination, 41,122 (44.6%) as moderate procrastination, 26,818 (29.1%) as high procrastination, and 5415 (5.9%) as very high/chronic procrastination.

Baseline demographic, lifestyle, anthropometric, hemodynamic, and cardiometabolic characteristics of the study population according to behavioral procrastination category are summarized in Table 1.

Higher behavioral procrastination levels were associated with a progressively less favorable cardiovascular and lifestyle profile, including older age, greater adiposity, higher blood pressure, poorer metabolic parameters, lower physical activity, and lower adherence to the Mediterranean diet.

Chronological age was strongly correlated with procrastination category (Pearson’s r = 0.669; *p* < 0.001), confirming the marked age gradient observed across procrastination groups.

To evaluate whether these marked differences were explained primarily by age distribution across procrastination categories, additional age-standardized descriptive analyses were performed and are presented in Table 2.

After age standardization, substantial gradients across procrastination categories persisted for adiposity, blood pressure, glucose, triglycerides, smoking, physical activity, and Mediterranean diet adherence. These findings indicate that the observed differences were not explained exclusively by the older age distribution of participants with higher procrastination levels. Coding of procrastination categories, smoking status, physical activity, and diet adherence was rechecked against the original database. The very low procrastination group included only three current smokers, corresponding to 0.02% of that category, which explains the apparently null prevalence reported in the rounded descriptive table.

### 3.2. Cardiovascular Aging Profile Across Procrastination Categories

Estimated heart age and heart age acceleration according to behavioral procrastination category are presented in Table 3.

Mean estimated heart age increased from 29.9 ± 8.9 years in participants with very low procrastination to 37.7 ± 10.7 years in those with moderate procrastination, 56.9 ± 11.6 years in those with high procrastination, and 70.4 ± 7.7 years in those with very high/chronic procrastination.

Mean heart age acceleration was −3.1 ± 6.0 years in the very low procrastination group, 2.7 ± 7.9 years in the moderate group, 9.1 ± 8.8 years in the high group, and 14.0 ± 6.4 years in the very high/chronic group.

### 3.3. Prevalence of Accelerated Cardiovascular Aging

**Figure 2:**
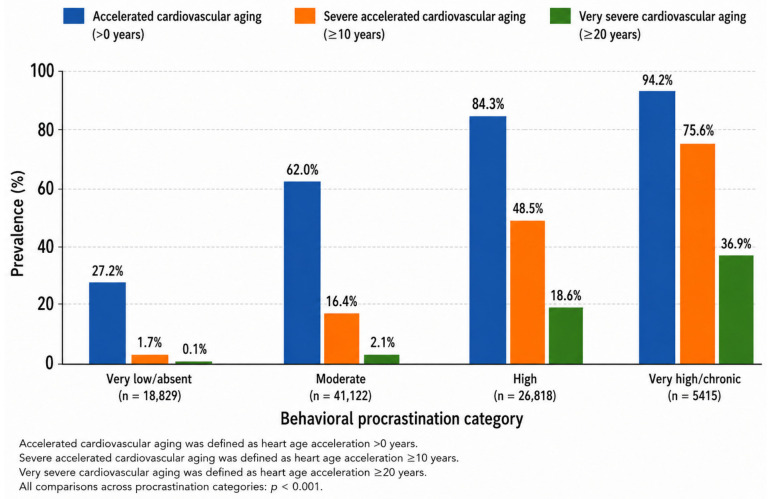
Prevalence of accelerated cardiovascular aging according to behavioral procrastination category.

The prevalence of accelerated cardiovascular aging phenotypes according to procrastination category is shown in Figure 2.

Accelerated cardiovascular aging, defined as heart age acceleration >0 years, was present in 27.2% of participants with very low procrastination, 62.0% of those with moderate procrastination, 84.3% of those with high procrastination, and 94.2% of those with very high/chronic procrastination.

Severe accelerated cardiovascular aging (≥10 years) was observed in 1.7%, 16.4%, 48.5%, and 75.6% of participants across increasing procrastination categories.

### 3.4. Association Between Procrastination Score and Heart Age Acceleration

Multivariable linear regression analyses evaluating the association between procrastination score and continuous heart age acceleration are presented in Table 4. To minimize mathematical coupling between predictors and outcome, variables directly incorporated into the heart age equation were excluded from the final adjusted regression model whenever appropriate.

In unadjusted analyses, each 5-point increase in procrastination score was associated with a 2.13-year increase in heart age acceleration (B = 2.13; 95% CI 2.09–2.17; *p* < 0.001).

After additional adjustment for BMI and cardiometabolic covariates not directly overlapping with the cardiovascular age algorithm, the association remained statistically significant. Each 5-point increase in procrastination score was associated with a 0.50-year increase in heart age acceleration (B = 0.50; 95% CI 0.48–0.52; *p* < 0.001).

### 3.5. Odds of Accelerated Cardiovascular Aging

Because accelerated cardiovascular aging was highly prevalent in this cohort, the ORs should be interpreted as measures of association rather than as estimates of prevalence ratios or relative risks. Therefore, complementary Poisson regression models with robust variance were fitted to estimate prevalence ratios (Table 5).

Compared with participants with very low procrastination, the adjusted odds of accelerated cardiovascular aging increased progressively across procrastination categories (Table 5). Because the outcome was common, complementary prevalence ratios estimated using Poisson regression are presented in Table 6 to facilitate interpretation of the magnitude of the associations.

A similar pattern was observed for severe accelerated cardiovascular aging. In fully adjusted models, compared with the very low procrastination group, the adjusted ORs were 1.40 (95% CI 1.24–1.58) for moderate procrastination, 1.73 (95% CI 1.53–1.97) for high procrastination, and 2.51 (95% CI 2.16–2.92) for very high/chronic procrastination.

To address the potential overestimation associated with odds ratios when outcome prevalence is high, additional Poisson regression models with robust variance were performed. Because accelerated cardiovascular aging was common in the study population, Poisson regression with robust variance was additionally performed to estimate prevalence ratios, which are more readily interpretable than ORs for common outcomes. The resulting PRs showed the same dose–response pattern observed in the logistic regression analyses, supporting the robustness of the findings while avoiding the potential overestimation inherent to ORs when outcome prevalence is high (Table 6).

### 3.6. Adjusted Association Between Behavioral Procrastination and Heart Age Acceleration

**Figure 3:**
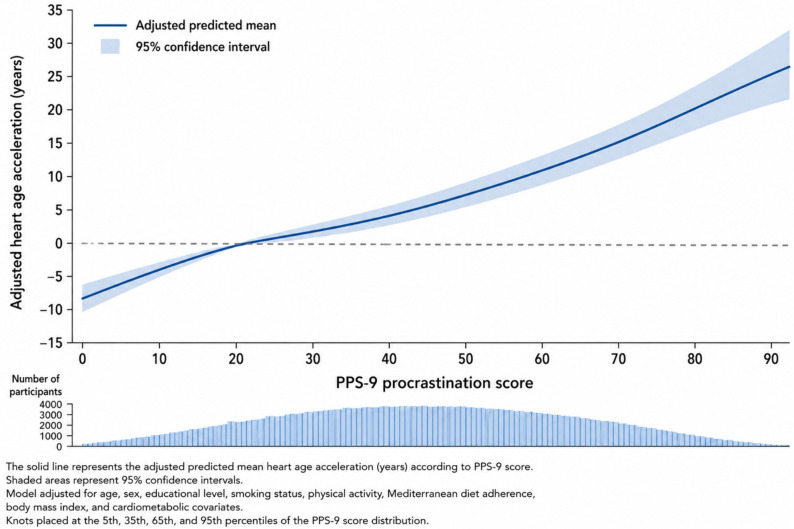
Adjusted association between behavioral procrastination score and heart age acceleration.

Adjusted analyses demonstrated a graded association between procrastination score and heart age acceleration (Figure 3).

Figure 3 shows the adjusted association between behavioral procrastination and heart age acceleration using restricted cubic spline regression. To facilitate interpretation and graphical visualization, PPS-9 scores were transformed into percentiles of their distribution within the study population. Consequently, the horizontal axis represents the percentile position of each participant’s procrastination score rather than the original PPS-9 score (9–45 points). For example, a value of 50 on the *x*-axis corresponds approximately to the median procrastination level of the sample, whereas values close to 0 and 100 represent the lowest and highest procrastination levels, respectively.

The solid blue line represents the adjusted predicted mean heart age acceleration, while the shaded area indicates the 95% confidence interval. The dashed horizontal line corresponds to a heart age acceleration of zero, indicating concordance between estimated heart age and chronological age.

The curve demonstrates a progressive and non-linear increase in heart age acceleration as procrastination levels rise. Participants located at the lower percentiles of the PPS-9 distribution tend to exhibit negative or near-zero values of heart age acceleration, whereas those at higher percentiles show increasingly positive values, indicating that their estimated cardiovascular age exceeds their chronological age. The steepening of the curve at the upper percentiles suggests that the adverse association between procrastination and cardiovascular aging becomes more pronounced among individuals with the highest levels of procrastination.

The histogram displayed below the *x*-axis shows the distribution of participants across PPS-9 percentiles, indicating that the estimates are based on a large number of observations in the central portion of the distribution and on fewer observations at the extremes.

### 3.7. Sex-Stratified Analyses

Sex-stratified multivariable analyses evaluating the association between behavioral procrastination score and heart age acceleration are presented in Table 7.

Among men, each 5-point increase in procrastination score was associated with a 0.51-year increase in heart age acceleration after multivariable adjustment (B = 0.51; 95% CI 0.48–0.54; *p* < 0.001).

Among women, each 5-point increase in procrastination score was associated with a 1.16-year increase in heart age acceleration after multivariable adjustment (B = 1.16; 95% CI 1.10–1.22; *p* < 0.001).

The sex interaction was statistically significant (*p* < 0.001), indicating a stronger association among women.

**Figure 4:**
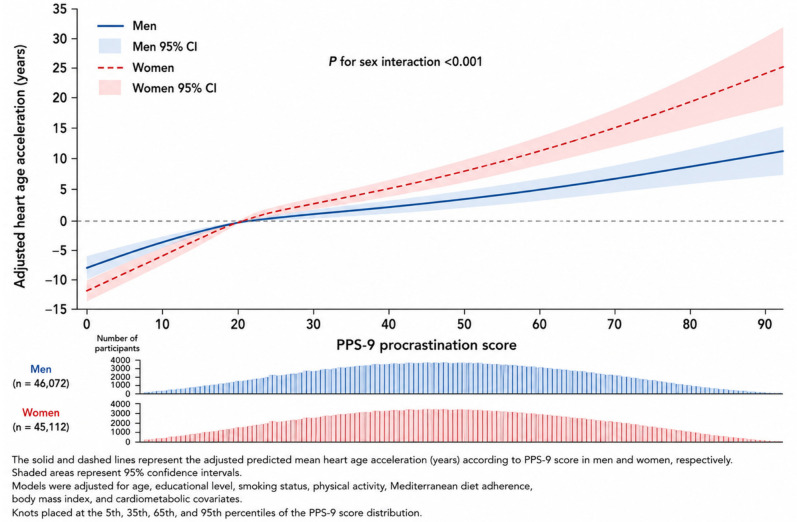
Sex-stratified dose–response relationship between behavioral procrastination and heart age acceleration.

Sex-specific dose–response associations between behavioral procrastination score and heart age acceleration are illustrated in Figure 4.

Figure 4 illustrates the adjusted association between behavioral procrastination and heart age acceleration separately for men and women. To facilitate visualization of the nonlinear association, the original PPS-9 total scores (range: 9–45 points) were transformed into percentiles based on their distribution in the study population. Consequently, the *x*-axis represents the relative position of participants within the procrastination score distribution rather than the original PPS-9 values. Lower percentiles correspond to individuals with lower levels of procrastination, whereas higher percentiles represent those with progressively greater procrastination.

The solid blue line (men) and dashed red line (women) represent the adjusted predicted mean heart age acceleration estimated from restricted cubic spline models, while the shaded areas indicate the corresponding 95% confidence intervals. All models were adjusted for age, educational level, smoking status, physical activity, adherence to the Mediterranean diet, body mass index, and cardiometabolic covariates. Knots were placed at the 5th, 35th, 65th, and 95th percentiles of the PPS-9 distribution.

In both sexes, heart age acceleration increased progressively with increasing levels of procrastination. However, the increase was substantially steeper among women than among men, indicating a stronger association between procrastination and cardiovascular aging in women. This difference was supported by a statistically significant sex interaction (*p* for interaction < 0.001). At lower percentiles of the PPS-9 distribution, both men and women exhibited negative values of heart age acceleration, whereas at higher percentiles the predicted values became progressively positive, with women reaching markedly higher levels than men.

### 3.8. Sensitivity Analyses

Sensitivity analyses excluding participants with diabetes mellitus are summarized in Table 8.

After excluding diabetic participants, the association between procrastination score and heart age acceleration remained robust. In the fully adjusted model (Model 3), each 5-point increase in procrastination score was associated with a 0.69-year increase in heart age acceleration (B = 0.69; 95% CI 0.66–0.72; *p* < 0.001).

### 3.9. Age-Stratified Sensitivity Analyses

Because chronological age differed markedly across procrastination categories, raising the possibility of residual age-related confounding, we performed additional age-stratified analyses to evaluate the robustness of the observed associations. Participants were categorized into four predefined age groups (18–34, 35–44, 45–54, and ≥55 years), and the relationship between procrastination and heart age acceleration was examined separately within each age stratum. The results of these analyses are presented in Table 9.

The association between procrastination and heart age acceleration remained statistically significant across all age groups, indicating that the observed findings were not solely explained by differences in chronological age between procrastination categories.

These findings indicate that the association between behavioral procrastination and heart age acceleration persisted within strata of comparable chronological age, supporting that the observed relationship was not explained exclusively by age-related clustering of procrastination categories.

## 4. Discussion

### 4.1. Principal Findings

In this large occupational cohort of more than 92,000 Spanish workers, higher levels of behavioral procrastination were consistently associated with less favorable cardiovascular aging profiles. Participants with greater procrastination severity exhibited substantially higher estimated heart age, greater heart age acceleration, and markedly increased prevalence of accelerated cardiovascular aging phenotypes. Importantly, these associations persisted after adjustment for demographic characteristics, lifestyle-related variables, adiposity indices, and cardiometabolic covariates, suggesting that behavioral procrastination may be correlated with less favorable cardiovascular aging phenotypes. However, the cross-sectional design does not allow determination of whether procrastination precedes cardiovascular aging or whether poorer health status may itself influence procrastination behaviors.

An important methodological consideration is that Heart Age is a composite metric derived from established cardiometabolic risk factors, including age, blood pressure, smoking status, diabetes status, body mass index, and lipid parameters. Several of these components may themselves be associated with behavioral procrastination through shared lifestyle and health-related pathways. Consequently, part of the observed association between procrastination and Heart Age acceleration may reflect these underlying cardiometabolic relationships rather than a direct effect of procrastination itself. The findings should therefore be interpreted as evidence of correlation rather than an independent causal relationship.

Several findings deserve particular attention. First, the magnitude of the observed gradients across procrastination categories was considerable. Participants with very high/chronic procrastination showed mean heart age acceleration values exceeding 14 years, whereas individuals with very low procrastination demonstrated cardiovascular profiles younger than their chronological age. Although the fully adjusted regression coefficient indicated an increase of approximately 0.50 years in heart age acceleration for every 5-point increase in PPS-9 score, this estimate should be interpreted in the context of population health rather than individual clinical decision-making. While the absolute effect size may appear modest at the individual level, behavioral procrastination is highly prevalent and the cumulative impact of small shifts in cardiovascular aging across large populations may still be clinically relevant from a public health perspective. Second, dose–response analyses revealed a progressive increase in cardiovascular aging burden across the entire procrastination continuum rather than a threshold effect restricted to extreme procrastination levels. Third, sex-stratified analyses suggested a substantially stronger association among women, supporting the possibility of sex-specific biological or psychosocial pathways linking self-regulatory dysfunction and cardiovascular aging.

Importantly, because substantial age differences were observed across procrastination categories and the correlation between procrastination score and age was relatively strong (r = 0.669), additional age-stratified analyses were performed. The association between procrastination and heart age acceleration remained statistically significant within all age groups, suggesting that age differences alone do not fully explain the observed findings. Nevertheless, residual age-related confounding cannot be completely excluded. Age may influence both procrastination behaviors and cardiovascular aging through complex biological, behavioral, occupational, and generational mechanisms that may not be fully accounted for by statistical adjustment or stratification. Therefore, the observed associations should be interpreted cautiously, and future longitudinal studies are needed to better disentangle the independent effects of age and procrastination on cardiovascular aging.

To our knowledge, this is one of the first large-scale epidemiological studies specifically evaluating the relationship between behavioral procrastination and objective cardiovascular aging phenotypes. Previous procrastination research has predominantly focused on academic performance, mental health, stress perception, or subjective well-being outcomes, whereas evidence linking procrastination with cardiovascular risk biomarkers has remained limited and fragmented [38,39,40,41].

### 4.2. Comparison with Previous Literature

The present findings are broadly consistent with previous evidence reporting associations between self-regulatory dysfunction, adverse health behaviors, and cardiometabolic risk accumulation. Sirois and colleagues previously proposed that procrastination may operate as a chronic stressor characterized by repeated cycles of avoidance, guilt, emotional dysregulation, and delayed health-promoting behaviors, ultimately contributing to poorer physical health outcomes [41]. Subsequent longitudinal studies have demonstrated associations between procrastination and increased stress burden, sleep disturbances, fatigue, unhealthy dietary behaviors, and lower engagement in preventive health behaviors [38,39,40,42].

Although direct evidence regarding procrastination and cardiovascular aging remains scarce, several studies support the plausibility of the associations observed in our analysis. Delayed healthcare seeking, poorer medication adherence, reduced physical activity, and higher sedentary behavior have all been associated with elevated procrastination traits and have also been linked to vascular dysfunction and adverse cardiometabolic profiles in previous studies [42,43]. Moreover, recent behavioral medicine research has increasingly emphasized that chronic maladaptive coping patterns may exert physiological effects through persistent activation of stress-related neuroendocrine pathways [44].

The observed relationship between procrastination and heart age acceleration may also align with broader literature evaluating psychosocial determinants of cardiovascular aging. Chronic psychological stress, emotional exhaustion, and impaired stress coping capacity have previously been associated with arterial stiffness, endothelial dysfunction, systemic inflammation, autonomic imbalance, and increased biological aging markers [45,46,47]. Similarly, adverse psychosocial exposures such as loneliness, occupational strain, sleep impairment, and depressive symptoms have demonstrated significant associations with accelerated vascular aging trajectories and excess cardiovascular mortality [48,49,50].

Our findings also reinforce emerging evidence suggesting that cardiovascular aging cannot be fully explained by classical biomedical risk factors alone. Increasingly, behavioral and psychosocial dimensions are recognized as important contributors to long-term cardiovascular vulnerability [51]. In this context, procrastination may represent a measurable behavioral phenotype reflecting chronic self-regulatory dysfunction, impaired future-oriented decision-making, and persistent maladaptive stress responses, all of which have been associated with cardiovascular aging in previous studies.

### 4.3. Potential Mechanisms

Several biological and behavioral mechanisms have been proposed in the previous literature and may be consistent with the associations observed in the present study. However, the cross-sectional design does not allow determination of whether these mechanisms are causally involved.

First, procrastination has been strongly linked to maladaptive lifestyle behaviors known to promote cardiometabolic dysfunction. Individuals with elevated procrastination levels frequently exhibit lower physical activity, poorer dietary quality, greater sleep irregularity, and reduced adherence to preventive health behaviors [38,39,40,41,42,43]. In our cohort, higher procrastination categories were consistently associated with lower Mediterranean diet adherence, reduced physical activity, greater adiposity, and less favorable metabolic profiles, supporting the possibility that lifestyle-related factors may be involved in the observed association.

Second, chronic emotional dysregulation may represent a central mechanistic pathway. Contemporary theoretical models conceptualize procrastination primarily as an emotion-regulation failure rather than a simple time-management problem [44]. Recurrent cycles of avoidance, stress anticipation, guilt, and self-criticism has been hypothesized to be associated with chronic activation of the hypothalamic–pituitary–adrenal axis and sympathetic nervous system, promoting inflammation, oxidative stress, endothelial dysfunction, visceral adiposity accumulation, and vascular remodeling [45,46].

Third, procrastination-related behaviors may contribute to cumulative allostatic load. Allostatic overload reflects the long-term physiological consequences of repeated stress adaptation and has been associated with accelerated biological aging and increased cardiovascular risk [47]. Individuals with chronic procrastination patterns may report persistent psychological distress and repeated failures in self-regulation, factors that have previously been associated with adverse physiological stress responses over prolonged periods.

The stronger associations observed among women are also noteworthy. Previous studies have suggested that women may exhibit greater cardiovascular sensitivity to psychosocial stressors, emotional burden, and chronic stress-related neuroendocrine activation [48,49]. Sex differences in emotional processing, stress coping strategies, autonomic regulation, and inflammatory responses may partially explain the steeper cardiovascular aging gradients observed among female workers in our cohort.

However, the present dataset did not include information on menopausal status, hormone replacement therapy, pregnancy history, caregiving responsibilities, depressive symptoms, perceived stress, or other sex-specific psychosocial factors. Therefore, the mechanisms underlying the stronger associations observed among women could not be directly evaluated and should be investigated in future studies.

### 4.4. Clinical and Public Health Implications

The present findings may have important implications for preventive cardiovascular medicine and occupational health strategies. Behavioral procrastination is highly prevalent in modern populations and is frequently normalized or underestimated in clinical practice. However, our results indicate that higher procrastination levels were associated with a greater cardiovascular aging burden. Given the cross-sectional design, it remains unclear whether procrastination precedes cardiovascular aging or whether poorer health status may influence procrastination behaviors. Accordingly, the observed dose–response relationships should be interpreted as hypothesis-generating associations rather than evidence of a confirmed biological mechanism or causal pathway.

Heart age models are increasingly used as communication tools for cardiovascular risk prevention because they facilitate individualized risk perception and may improve motivation for behavioral change [6]. Although procrastination was associated with cardiovascular aging phenotypes in the present study, further longitudinal research is required before considering its incorporation into cardiovascular risk assessment frameworks.

From an occupational health perspective, these findings are particularly relevant. Contemporary work environments often combine sedentary behavior, psychological stress, time pressure, irregular sleep schedules, and unhealthy lifestyle habits, all of which may interact synergistically with maladaptive self-regulatory behaviors [50]. Whether interventions targeting procrastination, emotional regulation, or related behavioral factors may influence cardiovascular aging remains unknown and should be evaluated in prospective intervention studies.

Importantly, procrastination should not be interpreted solely as an individual personality weakness. Increasing evidence suggests that procrastination emerges from complex interactions between emotional regulation, environmental stressors, executive functioning, and behavioral reinforcement mechanisms [44]. Consequently, multidimensional preventive approaches integrating psychological, behavioral, and occupational interventions may be necessary to effectively reduce associated health risks.

### 4.5. Strengths and Limitations

This study has several important strengths. The large sample size provided substantial statistical power and allowed robust evaluation of dose–response relationships and sex-specific associations. The occupational setting enabled standardized assessment of anthropometric, biochemical, hemodynamic, and behavioral variables under relatively homogeneous clinical conditions. Furthermore, the use of cardiovascular aging phenotypes rather than isolated risk factors provided a more integrative perspective regarding cumulative cardiovascular risk burden.

Nevertheless, several limitations should also be acknowledged. First, the cross-sectional design precludes causal inference, and bidirectional relationships between procrastination and cardiovascular health cannot be excluded. In addition, 2143 workers were excluded because of missing information in one or more study variables. Because detailed characteristics of excluded individuals were not retained in the final analytical database, formal comparisons between included and excluded participants could not be performed. Consequently, the potential impact of selection bias related to missing data cannot be completely excluded. Participants with incomplete information may have differed systematically from those included in the final analytical sample with respect to demographic characteristics, health behaviors, procrastination levels, or cardiometabolic risk profiles. Therefore, the representativeness of the study population and the generalizability of the findings should be interpreted with appropriate caution. Accordingly, reverse causation remains a plausible explanation for the observed associations. It is possible that poorer cardiovascular health, obesity, fatigue, reduced physical functioning, or psychological distress may increase procrastination behaviors rather than procrastination preceding cardiovascular aging. Therefore, the temporal direction of the association cannot be established from the present data. Although age-stratified analyses confirmed the persistence of the association across all age groups, residual age-related confounding cannot be completely excluded because procrastination categories showed substantial differences in chronological age. In addition, the marked age gradient observed across procrastination categories may partly reflect generational or cohort-related differences in behavioral patterns, social norms, or work-related characteristics rather than a direct relationship between procrastination and cardiovascular aging. This possibility should be considered when interpreting the findings. Second, behavioral procrastination, physical activity, and Mediterranean diet adherence were assessed using self-reported questionnaires. Consequently, information bias resulting from recall errors, inaccurate reporting, or social desirability effects cannot be excluded. Because both the exposure (behavioral procrastination) and several lifestyle-related covariates were self-reported, common method bias may also have influenced the observed associations. Furthermore, some degree of non-differential misclassification of lifestyle behaviors is possible, which may have attenuated or distorted the magnitude of the reported associations. Moreover, objective measures of physical activity, sleep behavior, or other lifestyle characteristics were not available. Future studies incorporating wearable devices or other objective monitoring methods may help reduce measurement error and improve exposure assessment. Furthermore, because only the total PPS-9 score was available in the occupational health database, we were unable to re-evaluate internal consistency indices such as Cronbach’s alpha or McDonald’s omega, or to examine the factor structure of the scale within the present cohort. Therefore, psychometric properties relied on previously published validation studies rather than on a new validation performed in the current sample. Third, residual confounding associated with unmeasured psychosocial, socioeconomic, psychiatric, occupational, and clinical variables remains possible despite multivariable adjustment. Important potential confounders such as depression, anxiety, attention-deficit/hyperactivity disorder (ADHD), sleep quality, insomnia symptoms, alcohol consumption, income level, occupational stress, shift work, burnout, medication use, family history of cardiovascular disease, menopausal status, hormone replacement therapy, pregnancy history, caregiving burden, perceived stress, and personality traits were not available in the present dataset and therefore could not be included in the analyses. These factors may influence both procrastination behaviors and cardiovascular health and should be considered in future studies.

Furthermore, some variables included in the multivariable models, such as physical activity, Mediterranean diet adherence, adiposity measures, and cardiometabolic characteristics, may represent potential mediators rather than pure confounders. Therefore, adjustment for these variables may have attenuated the observed associations, and the present analyses should not be interpreted as formal mediation models. Because of the cross-sectional design, temporal ordering between procrastination, lifestyle behaviors, cardiometabolic factors, and cardiovascular aging could not be established.

In addition, the progressive attenuation of the regression coefficient across sequentially adjusted models suggests that part of the crude association was explained by demographic, lifestyle, and cardiometabolic factors. However, the present analyses were designed to estimate adjusted associations rather than to formally quantify the relative contribution of individual covariate blocks. Therefore, partial R^2^ statistics and variance decomposition analyses were not performed and should be considered in future investigations.

Although specific measures were implemented to minimize mathematical coupling between predictors and outcome, complete independence between the cardiovascular age estimation process and all adjusted covariates cannot be guaranteed. Therefore, some degree of residual overlap between variables included in cardiovascular age estimation and those considered in multivariable analyses may persist.

Additionally, heart age was estimated using a validated Framingham-based cardiovascular risk algorithm rather than direct vascular imaging, arterial stiffness assessment, endothelial function testing, or other objective vascular biomarkers. Consequently, heart age should be interpreted as an indirect estimate of cardiovascular aging. Although heart age constitutes a clinically useful and intuitive cardiovascular aging metric, future longitudinal studies incorporating direct vascular biomarkers may help further clarify the biological mechanisms underlying these associations. Finally, the study population consisted exclusively of actively employed Spanish workers. Consequently, healthy worker bias cannot be excluded, as individuals who are unemployed, retired, on long-term sick leave, or affected by severe chronic illness were not represented in the study population. This limitation may have led to an underestimation of the overall burden of cardiovascular risk and restricts the generalizability of the findings to unemployed, retired, clinically vulnerable, or non-European populations.

Although age-stratified analyses demonstrated consistent associations across all age groups, the strong correlation between procrastination and age observed in this cohort (r = 0.669) indicates substantial age clustering. Consequently, some degree of residual age-related confounding may persist despite adjustment and stratification procedures. Generational differences in behavioral patterns, occupational characteristics, and health profiles may partly contribute to the observed associations.

An additional methodological consideration relates to the interpretation of effect measures. Because accelerated cardiovascular aging was a highly prevalent outcome, odds ratios may overestimate the magnitude of association compared with prevalence ratios. For this reason, complementary Poisson regression analyses with robust variance were performed. The consistency of the OR- and PR-based analyses strengthens the robustness of the observed associations while providing effect estimates that are more appropriate for common outcomes.

Another limitation relates to the nature of the Heart Age outcome itself. Because Heart Age is calculated from a combination of cardiometabolic risk factors that are also associated with behavioral procrastination, some degree of conceptual overlap between exposure-related behaviors and outcome components cannot be excluded. Although the statistical models adjusted for multiple demographic and lifestyle factors and sensitivity analyses were performed, the possibility that the observed associations partly reflect shared cardiometabolic pathways rather than a unique contribution of procrastination should be acknowledged.

Finally, variance decomposition analyses and partial R^2^ estimates were not performed. Consequently, the relative contribution of behavioral procrastination compared with other demographic, lifestyle, and cardiometabolic variables to the explained variability in heart age acceleration could not be quantified. Future studies specifically designed for predictive modeling may benefit from incorporating these approaches to better characterize the relative importance of individual predictors.

### 4.6. Future Directions

Future prospective studies are needed to determine the temporal sequence between procrastination and cardiovascular aging and to clarify whether procrastination precedes cardiovascular changes or emerges as a consequence of poorer health status. Research incorporating objective vascular biomarkers, inflammatory mediators, autonomic parameters, and stress-related neuroendocrine markers could help clarify the biological pathways linking procrastination with cardiovascular aging.

Additionally, intervention studies evaluating whether improvements in emotional regulation, behavioral self-management, sleep quality, or stress coping strategies can attenuate cardiovascular aging trajectories among individuals with elevated procrastination traits would be of considerable interest. Given the stronger associations observed among women, future investigations should also explore potential sex-specific psychosocial and biological mechanisms in greater detail.

## 5. Conclusions

Behavioral procrastination was associated with higher estimated heart age acceleration and less favorable cardiovascular aging profiles in this large occupational cohort of Spanish workers. Higher procrastination levels were consistently related to greater estimated heart age acceleration, a higher prevalence of cardiovascular aging phenotypes, and less favorable cardiometabolic profiles, even after extensive multivariable adjustment.

The observed dose–response relationship indicates that higher procrastination levels were associated with progressively less favorable cardiovascular aging profiles rather than being limited to individuals with extreme procrastination traits. Moreover, stronger associations were observed among women, suggesting the possibility of sex-specific psychosocial or behavioral correlates.

However, these findings should be interpreted with caution. Heart age is a composite measure derived from established cardiometabolic risk factors, several of which may themselves be associated with procrastination-related behaviors and lifestyle characteristics. Consequently, part of the observed association may reflect shared cardiometabolic and behavioral pathways rather than an independent effect of procrastination on cardiovascular aging.

The present results should therefore be considered hypothesis-generating and do not establish causal relationships, temporality, or specific biological mechanisms. Given the cross-sectional design, reverse causation remains a plausible alternative explanation, and residual confounding cannot be completely excluded.

Furthermore, because the study population was restricted to actively employed Spanish workers, caution is warranted when extrapolating these findings to other population groups.

Although behavioral procrastination was consistently associated with cardiovascular aging indicators, the observed associations do not support considering procrastination as a major determinant of cardiovascular risk. The magnitude of the fully adjusted associations was relatively modest at the individual level despite their statistical robustness. Therefore, procrastination may represent one of several behavioral characteristics associated with less favorable cardiovascular aging profiles rather than a major independent cardiovascular risk factor.

Behavioral procrastination should not currently be considered an established independent cardiovascular risk factor. Longitudinal studies incorporating direct vascular and subclinical cardiovascular biomarkers are required to clarify temporality, evaluate the independent contribution of procrastination to cardiovascular aging, and better characterize the biological and behavioral mechanisms underlying the observed associations.

## Figures and Tables

**Table 1:** Baseline characteristics of the study population according to behavioral procrastination category.

Variable	Very Low/Absent Procrastination	Moderate Procrastination	High Procrastination	Very High/Chronic Procrastination	*p*-Value
Participants, *n* (%)	18,829 (20.4)	41,122 (44.6)	26,818 (29.1)	5415 (5.9)	<0.001
Age, years	33.0 ± 6.5	39.1 ± 8.4	48.2 ± 8.7	56.4 ± 5.7	<0.001
Male sex, n (%)	8737 (46.4)	22,840 (55.5)	18,782 (70.0)	4122 (76.1)	<0.001
Body mass index, kg/m^2^	22.3 ± 1.8	25.4 ± 3.1	28.7 ± 4.0	31.0 ± 4.6	<0.001
Waist circumference, cm	75.2 ± 7.6	82.4 ± 8.9	87.9 ± 10.1	91.5 ± 11.2	<0.001
Waist-to-height ratio	0.44 ± 0.04	0.49 ± 0.05	0.54 ± 0.06	0.57 ± 0.07	<0.001
Systolic blood pressure, mmHg	113.0 ± 12.5	120.8 ± 14.6	128.3 ± 16.1	134.6 ± 18.2	<0.001
Diastolic blood pressure, mmHg	71.5 ± 8.3	76.9 ± 9.4	82.4 ± 10.6	86.9 ± 11.7	<0.001
Fasting glucose, mg/dL	87.2 ± 9.8	92.5 ± 11.4	99.1 ± 14.2	106.8 ± 18.5	<0.001
Triglycerides, mg/dL	92.6 ± 38.4	116.9 ± 51.3	141.5 ± 67.8	168.3 ± 82.7	<0.001
HDL-cholesterol, mg/dL	61.2 ± 12.4	56.3 ± 11.6	51.4 ± 10.8	47.1 ± 10.3	<0.001
Current smoker, *n* (%)	3 (0.02)	18,056 (43.9)	12,256 (45.7)	2306 (42.6)	<0.001
Regular physical activity, *n* (%)	15,970 (84.8)	24,468 (59.5)	8690 (32.4)	520 (9.6)	<0.001
High Mediterranean diet adherence, *n* (%)	14,741 (78.3)	20,355 (49.5)	7185 (26.8)	515 (9.5)	<0.001

Values are presented as mean ± standard deviation or absolute frequency and percentage, as appropriate. Behavioral procrastination categories were defined according to PPS-9 score distribution. *p*-values were obtained using one-way ANOVA for continuous variables and chi-square tests for categorical variables.

**Table 2:** Age-standardized descriptive characteristics according to behavioral procrastination category.

Variable	Very Low/Absent	Moderate	High	Very High/Chronic
BMI, kg/m^2^	22.38	26.19	29.72	35.00
Systolic blood pressure, mmHg	113.36	120.66	124.95	129.22
Fasting glucose, mg/dL	82.89	87.86	88.51	92.84
Triglycerides, mg/dL	81.54	100.57	142.19	158.89
HDL-cholesterol, mg/dL	55.17	51.88	49.80	51.35
Current smoker, %	0.01	53.48	66.09	81.15
Regular physical activity, %	83.42	56.79	17.95	1.40
High Mediterranean diet adherence, %	76.32	38.62	16.66	1.24

Values were age-standardized using the age distribution of the overall analytical sample as the standard population. BMI: body mass index; HDL: high-density lipoprotein.

**Table 3:** Cardiovascular aging profile according to behavioral procrastination category.

Variable	Very Low/Absent Procrastination	Moderate Procrastination	High Procrastination	Very High/Chronic Procrastination	*p*-Value
Estimated heart age, years	29.9 ± 8.9	37.7 ± 10.7	56.9 ± 11.6	70.4 ± 7.7	<0.001
Heart age acceleration, years	−3.1 ± 6.0	2.7 ± 7.9	9.1 ± 8.8	14.0 ± 6.4	<0.001
Accelerated cardiovascular aging (>0 years), n (%)	5122 (27.2)	25,496 (62.0)	22,607 (84.3)	5101 (94.2)	<0.001
Severe accelerated cardiovascular aging (≥10 years), n (%)	320 (1.7)	6744 (16.4)	13,006 (48.5)	4093 (75.6)	<0.001
Very severe cardiovascular aging (≥20 years), n (%)	19 (0.1)	864 (2.1)	4988 (18.6)	1998 (36.9)	<0.001

Values are presented as mean ± standard deviation or absolute frequency and percentage, as appropriate. Heart age acceleration was calculated as estimated heart age minus chronological age. Accelerated cardiovascular aging was defined as heart age acceleration >0 years. Severe accelerated cardiovascular aging was defined as heart age acceleration ≥10 years.

**Table 4:** Multivariable linear regression analyses for the association between behavioral procrastination score and heart age acceleration.

Model	β Coefficient	Unstandardized Coefficient (B)	95% CI	*p*-Value
Model 1: Unadjusted	0.589	2.13	2.09 to 2.17	<0.001
Model 2: Adjusted for age and sex	0.564	2.04	1.99 to 2.08	<0.001
Model 3: Additionally adjusted for educational level, smoking, physical activity, and Mediterranean diet adherence	0.200	0.72	0.69 to 0.75	<0.001
Model 4: Additionally adjusted for BMI and cardiometabolic covariates	0.139	0.50	0.48 to 0.52	<0.001

Heart age acceleration was calculated as estimated heart age minus chronological age. Behavioral procrastination score was analyzed as a continuous variable. Regression coefficients represent the increase in heart age acceleration associated with each 5-point increase in PPS-9 score. Model 1: unadjusted. Model 2: adjusted for age and sex. Model 3: additionally adjusted for educational level, smoking status, physical activity, and Mediterranean diet adherence. Model 4: additionally adjusted for educational level, smoking status, physical activity, Mediterranean diet adherence, waist-to-height ratio, and triglyceride concentration. Variables embedded within the heart age equation were not reintroduced into the model. β: standardized beta coefficient; B: unstandardized regression coefficient; CI: confidence interval.

**Table 5:** Adjusted odds ratios for accelerated cardiovascular aging according to behavioral procrastination category.

Behavioral Procrastination Category	Accelerated Cardiovascular Aging (>0 Years) OR (95% CI)	*p*-Value	Severe Accelerated Cardiovascular Aging (≥10 Years) OR (95% CI)	*p*-Value
Very low/absent procrastination	Reference	—	Reference	—
Moderate procrastination	1.15 (1.10–1.21)	<0.001	1.40 (1.24–1.58)	<0.001
High procrastination	1.37 (1.28–1.46)	<0.001	1.73 (1.53–1.97)	<0.001
Very high/chronic procrastination	1.89 (1.65–2.18)	<0.001	2.51 (2.16–2.92)	<0.001

Odds ratios (ORs) and 95% confidence intervals (95% CIs) were estimated using multivariable logistic regression analyses. Accelerated cardiovascular aging was defined as heart age acceleration >0 years. Severe accelerated cardiovascular aging was defined as heart age acceleration ≥10 years. Models were adjusted for age, sex, educational level, smoking status, physical activity, Mediterranean diet adherence, body mass index, and cardiometabolic covariates not directly overlapping with the cardiovascular age estimation algorithm. CI: confidence interval; OR: odds ratio.

**Table 6:** Prevalence ratios for accelerated cardiovascular aging according to behavioral procrastination category estimated using Poisson regression with robust variance.

Behavioral Procrastination Category	PR	95% CI	*p*-Value
Very low/absent procrastination	Reference	—	—
Moderate procrastination	2.28	2.23–2.34	<0.001
High procrastination	3.10	3.03–3.18	<0.001
Very high/chronic procrastination	3.47	3.38–3.55	<0.001

Prevalence ratios (PRs) and 95% confidence intervals were estimated using Poisson regression with robust variance. The very low/absent procrastination group was used as the reference category.

**Table 7:** Sex-stratified multivariable linear regression analyses for the association between behavioral procrastination score and heart age acceleration.

Sex	β Coefficient	Unstandardized Coefficient (B)	95% CI	*p*-Value
Men	0.165	0.51	0.48 to 0.54	<0.001
Women	0.276	1.16	1.10 to 1.22	<0.001
Sex interaction term	—	—	*p*-interaction < 0.001	—

Heart age acceleration was calculated as estimated heart age minus chronological age. Behavioral procrastination score was analyzed as a continuous variable. Models were adjusted for age, educational level, smoking status, physical activity, Mediterranean diet adherence, body mass index, and cardiometabolic covariates not directly overlapping with the cardiovascular age estimation algorithm. Regression coefficients represent the increase in heart age acceleration associated with each 5-point increase in PPS-9 score. β: standardized beta coefficient; B: unstandardized regression coefficient; CI: confidence interval.

**Table 8:** Sensitivity analyses excluding participants with diabetes mellitus.

Model	β Coefficient	Unstandardized Coefficient (B)	95% CI	*p*-Value
Model 1: Unadjusted	0.561	1.98	1.94 to 2.02	<0.001
Model 2: Adjusted for age and sex	0.533	1.84	1.80 to 1.88	<0.001
Model 3: Additionally adjusted for educational level, smoking, physical activity, and Mediterranean diet adherence	0.190	0.69	0.66 to 0.72	<0.001
Model 4: Additionally adjusted for BMI and cardiometabolic covariates	0.128	0.46	0.44 to 0.48	<0.001

Sensitivity analyses were performed after exclusion of participants with diabetes mellitus. Heart age acceleration was calculated as estimated heart age minus chronological age. Behavioral procrastination score was analyzed as a continuous variable. Regression coefficients represent the increase in heart age acceleration associated with each 5-point increase in PPS-9 score. Model 1: unadjusted. Model 2: adjusted for age and sex. Model 3: additionally adjusted for educational level, smoking status, physical activity, and Mediterranean diet adherence. Model 4: additionally adjusted for body mass index and cardiometabolic covariates not directly overlapping with the cardiovascular age estimation algorithm. β: standardized beta coefficient; B: unstandardized regression coefficient; CI: confidence interval.

**Table 9:** Age-stratified association between procrastination level and heart age acceleration.

Age Group	n	B Coefficient	95% CI	*p*-Value
18–34 years	31,649	5.28	5.18–5.38	<0.001
35–44 years	30,877	7.28	7.14–7.42	<0.001
45–54 years	21,549	8.41	8.17–8.64	<0.001
≥55 years	8109	4.18	3.81–4.55	<0.001

Regression coefficients represent the increase in heart age acceleration across increasing procrastination levels within each age stratum. Coefficients were obtained from Model 4 adjusted for sex, educational level, smoking status, physical activity, Mediterranean diet adherence, body mass index, and cardiometabolic covariates.

## Data Availability

The datasets generated and analyzed during the current study are maintained in a secure institutional repository at ADEMA University School. Access to fully anonymised data may be considered upon reasonable request to the corresponding author, subject to compliance with applicable ethical requirements and current data protection legislation. The data presented in this study are available on request from the corresponding author. Data are available from ADEMA University School upon reasonable request, subject to data protection restrictions.
